# Dysfunctional bone marrow endothelial progenitor cells are involved in patients with myelodysplastic syndromes

**DOI:** 10.1186/s12967-022-03354-2

**Published:** 2022-03-29

**Authors:** Tong Xing, Zhong-Shi Lyu, Cai-Wen Duan, Hong-Yan Zhao, Shu-Qian Tang, Qi Wen, Yuan-Yuan Zhang, Meng Lv, Yu Wang, Lan-Ping Xu, Xiao-Hui Zhang, Xiao-Jun Huang, Yuan Kong

**Affiliations:** 1 Peking University People’s Hospital, Peking University Institute of Hematology, National Clinical Research Center for Hematologic Disease, Beijing Key Laboratory of Hematopoietic Stem Cell Transplantation, Collaborative Innovation Center of Hematology, Peking University, Beijing, China; 2 Peking-Tsinghua Center for Life Sciences, Academy for Advanced Interdisciplinary Studies, Peking University, Beijing, China; 3 Key Laboratory of Pediatric Hematology and Oncology Ministry of Health and Pediatric Translational Medicine Institute, Shanghai Children’s Medical Center, Shanghai Collaborative Innovation Center for Translational Medicine and Department of Pharmacology and Chemical Biology, Shanghai Jiao Tong University School of Medicine, Shanghai, China

**Keywords:** Myelodysplastic syndromes, Endothelial progenitor cells, Haematopoiesis, Immune regulation

## Abstract

**Background:**

Myelodysplastic syndromes (MDS) are a group of heterogeneous myeloid clonal disorders characterized by ineffective haematopoiesis and immune deregulation. Emerging evidence has shown the effect of bone marrow (BM) endothelial progenitor cells (EPCs) in regulating haematopoiesis and immune balance. However, the number and functions of BM EPCs in patients with different stages of MDS remain largely unknown.

**Methods:**

Patients with MDS (N = 30), de novo acute myeloid leukaemia (AML) (N = 15), and healthy donors (HDs) (N = 15) were enrolled. MDS patients were divided into lower-risk MDS (N = 15) and higher-risk MDS (N = 15) groups according to the dichotomization of the Revised International Prognostic Scoring System. Flow cytometry was performed to analyse the number of BM EPCs. Tube formation and migration assays were performed to evaluate the functions of BM EPCs. In order to assess the gene expression profiles of BM EPCs, RNA sequencing (RNA-seq) were performed. BM EPC supporting abilities of haematopoietic stem cells (HSCs), leukaemia cells and T cells were assessed by in vitro coculture experiments.

**Results:**

Increased but dysfunctional BM EPCs were found in MDS patients compared with HDs, especially in patients with higher-risk MDS. RNA-seq indicated the progressive change and differences of haematopoiesis- and immune-related pathways and genes in MDS BM EPCs. In vitro coculture experiments verified that BM EPCs from HDs, lower-risk MDS, and higher-risk MDS to AML exhibited a progressively decreased ability to support HSCs, manifested as elevated apoptosis rates and intracellular reactive oxygen species (ROS) levels and decreased colony-forming unit plating efficiencies of HSCs. Moreover, BM EPCs from higher-risk MDS patients demonstrated an increased ability to support leukaemia cells, characterized by increased proliferation, leukaemia colony-forming unit plating efficiencies, decreased apoptosis rates and apoptosis-related genes. Furthermore, BM EPCs induced T cell differentiation towards more immune-tolerant cells in higher-risk MDS patients in vitro. In addition, the levels of intracellular ROS and the apoptosis ratios were increased in BM EPCs from MDS patients, especially in higher-risk MDS patients, which may be therapeutic candidates for MDS patients.

**Conclusion:**

Our results suggest that dysfunctional BM EPCs are involved in MDS patients, which indicates that improving haematopoiesis supporting ability and immuneregulation ability of BM EPCs may represent a promising therapeutic approach for MDS patients.

**Supplementary Information:**

The online version contains supplementary material available at 10.1186/s12967-022-03354-2.

## Background

Myelodysplastic syndromes (MDS) refer to a group of heterogeneous myeloid clonal disorders characterized by abnormal development of myeloid cells, which manifest as ineffective haematopoiesis and a tendency to transform into acute myeloid leukaemia (AML) [1–3]. The pathogenesis of MDS is complex and diverse and includes inherent genetic abnormalities in myeloid progenitor cells, changes in the bone marrow (BM) microenvironment, and chronic immune stimulation [1, 4, 5]. Current strategies to treat MDS rely on allogeneic haematopoietic stem cell transplantation (allo-HSCT), hypomethylating agents, immunosuppressive therapy and chemotherapy [6–9], which have not achieved satisfactory clinical efficacy [10, 11]. Taking allo-HSCT as an example, only 40 to 50% of patients survive for 5 years [12, 13]. Therefore, further study of MDS pathogenesis is urgently needed to provide new treatment strategies for MDS patients.

The growth and spread of malignant clones represent the dominant pathophysiological process of MDS [14, 15]. With disease progression, malignant cells gradually replace normal haematopoietic stem cells (HSCs) and eventually dominate the BM [16, 17]. Progression of MDS to AML is thought to result from a shift from apoptosis to proliferation of these malignant clones [18]. As another important pathogenic mechanism of MDS, immune deregulation has been shown in a great number of MDS patients [19, 20]. For example, T helper (Th) 17 cells were increased in lower-risk MDS, and regulatory T cells (Tregs) were increased in higher-risk MDS [21, 22], which suggests that progression is facilitated by immune suppression. Therefore, understanding the mechanisms of ineffective haematopoiesis and immune deregulation in MDS patients is of considerable importance.

Emerging evidence has shown important roles of the BM microenvironment in regulating haematopoiesis and immune balance [23–25]. Previous murine studies [24, 26, 27] and our previous studies [28–30] have reported that endothelial progenitor cells (EPCs) are an important component of the normal BM microenvironment to support HSCs. However, Sophia et al. reported that increased BM EPCs may contribute to inferior haematopoietic function in a MDS murine model [31]. In vitro experiments have shown a poor ability of endothelial colony forming cells from the peripheral blood of MDS patients to support HSCs [32]. These evidences suggest that EPCs demonstrate inferior supporting ability to normal HSCs in MDS whereas the supporting abilities in patients with different stages of MDS remain to be comprehensively depicted. In terms of malignant cells, intrinsic apoptotic signals like clonal chromosomal changes or gene mutations contribute to progression to AML [16]. However, extrinsic apoptotic signals which are related to immune and microenvironment are also considered the pivotal reasons of the transformation of clonal progenitors from MDS to AML [1]. Mice transplantation experiment proved that leukemia-derived endothelial cells are capable of giving rise to AML in normal mice [33]. However, the supporting abilities of BM EPCs to malignant cells in MDS remain unclear. Furthermore, although normal human EPCs exhibit immunosuppressive properties [34–36], the immunomodulatory effect of BM EPCs has not been reported in MDS patients.

Therefore, the current study was performed to explore the functions of BM EPCs in MDS patients, including lower-risk MDS and higher-risk MDS patients. The number and functions of BM EPCs from MDS patients, de novo AML patients and healthy donors (HDs) were analysed. Bulk RNA sequencing (RNA-seq) was used to further explore the expression profile of BM EPCs, and in vitro coculture strategies were used to evaluate the abilities of BM EPCs to regulate haematopoiesis and immunity. Our aim was to provide a potential therapeutic strategy for MDS patients.

## Methods

### Patients and controls

Patients with MDS (N = 30) and de novo AML (N = 15) were enrolled. Newly diagnosed MDS patients (N = 30) were divided into lower-risk MDS (N = 15) and higher-risk MDS (N = 15) groups (Additional file 1: Table S1) according to the dichotomization of the Revised International Prognostic Scoring System (IPSS-R) [37]. AML patients were diagnosed with de novo M2 (N = 8), M4 (N = 2) or M5 (N = 5) disease [38]. Bone marrow cells from allogenetic transplantation donors (N = 15) were used as normal controls. The age of lower-risk MDS (51.3 years, range 29–69), higher-risk MDS (48.4 years, range 24–68), AML (51.2 years, range 26–64) patients and HDs (49.5 years, range 29–64) showed no significant differences.

### Isolation, cultivation, and characterization of primary BM EPCs

As previously reported [28–30, 39, 40], BM mononuclear cells (BMMNCs) were isolated by density gradient centrifugation using lymphocyte separation medium (GE Healthcare, Milwaukee, USA). Precultivated BM EPCs were characterized by staining with mouse anti-human CD45 (BD Biosciences, San Jose, USA), CD34 (BioLegend, San Diego, USA), CD133 (Miltenyi Biotec, Bergisch Gladbach, Germany), and vascular endothelial growth factor receptor 2 (VEGFR2, CD309) monoclonal antibodies (BD Biosciences, San Jose, USA). Data were analysed with BD FACSDIVA v8.0 Software (BD Biosciences).

BMMNCs (5 × 10^6^ per well) were cultured in fibronectin-precoated (Sigma, St. Louis, USA) 6-well culture plates with EGM-2-MV-SingleQuots (Lonza, Walkersville, USA) and 10% foetal bovine serum (FBS; Gibco, Australia) at 37 °C in a humidified incubator with 5% CO_2_ for 7 days until testing.

BM adherent cells at day 7 of cultivation were further functionally characterized as BM EPCs for their capacity to uptake Acetylated low-density lipoprotein (Ac-LDL) and to bind Ulex europaeus agglutinin I (UEA I) [28–30, 40, 41]. The adherent cells were incubated with DiI-AcLDL (Life Technologies, Gaithersburg, USA) at 37 °C. After 4 h, the cells were fixed with 4% prechilled paraformaldehyde and incubated with 10 μg/ml fluorescein isothiocyanate-labeled-labelled UEA I (FITC-UEA I; Sigma, St. Louis, USA) for 1 h. To evaluate the numbers of double-positive-stained EPCs, three power fields were randomly counted using a fluorescence microscope (Olympus, Tokyo, Japan).

After 7 days of cultivation, EPC identity was confirmed by real-time quantitative polymerase chain reaction (qRT-PCR) for endothelial specific marker genes (VEGFR2, VE-cadherin and vWF) [41, 42].

### Tube formation and migration assays

A total of 4 × 10^4^ EPCs at day 7 of cultivation were transferred to matrigel-coated (Corning, New York, USA) plates and incubated for 48 h at 37 °C in 5% CO_2_. Tube formation [28, 29, 39, 40] was measured by determining the relative tube length per field of view using an inverted light microscope. All cells were counted in three random fields.

Cell migration [28–30, 39, 40, 43] was assayed using a transwell chamber (Corning, New York, USA). The EPCs after 7 days of culture were seeded in the upper chambers at a density of 4 × 10^4^ cells per well, while 500 μl medium was added to the lower chamber. The cells were cultured for 24 h, and migrated cells were fixed with paraformaldehyde for 30 min. Then, cells on the bottom surface of the membrane were stained with crystal violet for 20 min and counted manually in three random fields/sample. Cell images were obtained on a phase-contrast microscope (Olympus, Tokyo, Japan).

### HSCs, T cells or HL-60 cells were cocultured with BM EPCs

In order to evaluate the effect of supporting ability of BM EPCs to normal HSCs, T cells or HL-60 cells, the cultivated BM EPCs from HDs or MDS or AML patients were cocultured with normal CD34^+^ cells, or normal CD3^+^ cells, or HL-60 cells [28–30, 40, 44]. HSCs or T cells were isolated from BMMNCs of HDs using CD34 or CD3 MicroBead kits (Miltenyi Biotec, Bergisch Gladbach, Germany). Cultivated EPCs (described before) were plated onto gelatinized 24-well culture plates at 1 × 10^5^ cells/well and cultured overnight to achieve confluence. Then, HSCs (1 × 10^5^ per well) or T cells (1 × 10^5^ per well) or HL-60 cells (Manassas, Virginia, USA; 5 × 10^4^ per well) were added in direct contact with confluent and adherent EPCs. EPC-HSC cocultures were maintained in StemSpan™ SFEM (Stem Cell Technologies, Vancouver, Canada) for 5 days. EPC-T cells or HL-60 cells cocultures were maintained in RPMI 1640 medium supplemented with 10% FBS for 3 days or 5 days. Appropriate controls of T cell culture alone were also included.

### Intracellular reactive oxygen species (ROS) levels

To detect ROS levels of HSCs and precultivated EPCs [28, 29, 40], HSCs were stained with CD34, and BMMNCs were stained with the aforementioned EPC markers and incubated with 10 μM 2ʹ,7ʹ-dichlorofluorescein diacetate (DCFH-DA, Byotime, Shanghai, China) at 37 °C for 15 min. All data were analysed on BD FACSDIVA v8.0 Software (BD Biosciences).

To detect ROS levels on day 7 of cultivated BM EPCs, adherent cells were incubated with 1 μg/ml DCFH-DA at 37 °C for 20 min. Images were obtained in three random fields/sample using a fluorescence microscope (Olympus, Tokyo, Japan). The fluorescence intensity of ROS was analysed via the mean grey value using ImageJ 1.52v (National Institutes of Health, Bethesda, USA).

### Apoptosis ratio analysis

To detect the apoptosis ratio, HSCs, precultivated EPCs or HL-60 cells were incubated with Annexin-V (BioLegend, San Diego, USA) and 7-amino-actinomycin D (7-AAD; BD Biosciences, San Jose, USA) for 10 min at room temperature and then analysed on BD FACSDIVA v8.0 Software (BD Biosciences).

### Colony-forming unit (CFU) and leukaemia colony-forming unit (CFU-L) assays

CFUs were assayed using MethoCult™ H4434 Classic (Stem Cell Technologies, Vancouver, Canada). After 5 days of coculture, 2 × 10^3^ CD34^+^ cells or 2 × 10^3^ HL-60 cells were plated in 24-well plates and cultured for 14 days. Colony-forming unit erythroid (CFU-E), burst-forming unit erythroid (BFU-E), colony-forming unit-granulocyte/macrophages (CFU-GM), and colony-forming unit-granulocyte, erythroid, macrophage and megakaryocyte (CFU-GEMM) measurements for CD34^+^ cells [28, 30, 44] and CFU-L measurements for HL-60 cells [45] were scored.

### Analysis of T cell subsets

After 3 days of coculture, CD3^+^ T cells were stimulated with a cell stimulation cocktail (eBioscience, San Diego, USA) to induce CD3^+^ T cell activation and cytokine secretion. Lymphocyte subpopulations were quantified via flow cytometry as previously described [46–49]. Th1, Th2, Th17 cells, and Tregs were identified as CD3^+^CD8^−^IFN-γ^+^, CD3^+^CD8^−^IL-4^+^, CD3^+^CD8^−^IL-17A^+^, and CD3^+^CD8^−^CD25^+^Foxp3^+^ populations, respectively. The details of antibodies were in Additional file 1: Table S2.

### 5-Ethynyl-20-deoxyuridine (EdU) assay

HL-60 cells were harvested after coculture with EPCs and then incubated with 50 μM EdU (RiboBio, Guangzhou, China) in 48-well plates for 1 h at 37 °C. Then, according to the manufacturer’s instructions, the nuclear fluorescence intensity was analysed on BD FACSDIVA v8.0 Software (BD Biosciences).

### RNA-seq and data analysis

RNA-seq analyses were performed to analyse the 7-day cultivated BM EPCs from HDs, lower-risk MDS, higher-risk MDS or AML patients. The accession number of whole transcriptome RNA-seq data is GSE197907. Differential gene expression (DEGs), principal component analysis (PCA), hierarchical clustering analysis and Kyoto Encyclopedia of Genes and Genomes (KEGG) enrichment plot were executed by the DESeq2, clusterProfiler, pheatmap, and ggplot2 packages in R (1.16.1). The top 20 up and down regulated genes in higher-risk MDS BM EPCs than lower-risk MDS BM EPCs were list in Additional file 1: Table S3 and in AML BM EPCs than higher-risk MDS BM EPCs were list in Additional file 1: Table S4. Gene set enrichment analysis (GSEA) analysis was performed with respect to MSigDB (version 5.1) genesets C5 GO biological processes [50].

### qRT-PCR

For qRT-PCR, RNA was extracted using the RNeasy Mini kit (QIAGEN, Dusseldorf, Germany). One microgram of RNA was reverse transcribed into cDNA by the RT reagent Kit with gDNA Eraser (TaKaRa, Otsu, Japan). The mRNA levels of *VEGFR2*, *VE-cadherin*, *VWF*, *CASP2*, *CASP3*, *BAX*, *CCNE1*, *MCL1*, *TP53*, *CDKN1A*, *CXCL12*, *KITLG*, *NFKB1*, *HAVCR2*, *CIITA*, and *LGALS9* were detected by the SYBR-Green qRT-PCR kit (Thermo Fisher Scientific, Waltham, USA). The levels of the aforementioned genes were evaluated after normalization to the *18S* mRNA level [30]. All sequences of primers were list in Additional file 1: Table S5.

### Statistical analysis

Analyses were performed using GraphPad Prism 6.0. Statistical analyses were performed using Mann–Whitney U test. The relative mRNA analyses and paired analyses were using Wilcoxon matched-pairs signed rank test. The results are expressed as the means ± SEM, and *P-*values < 0.05 were considered statistically significant.

## Results

### Increased number of BM EPCs in MDS patients

**Fig. 1:**
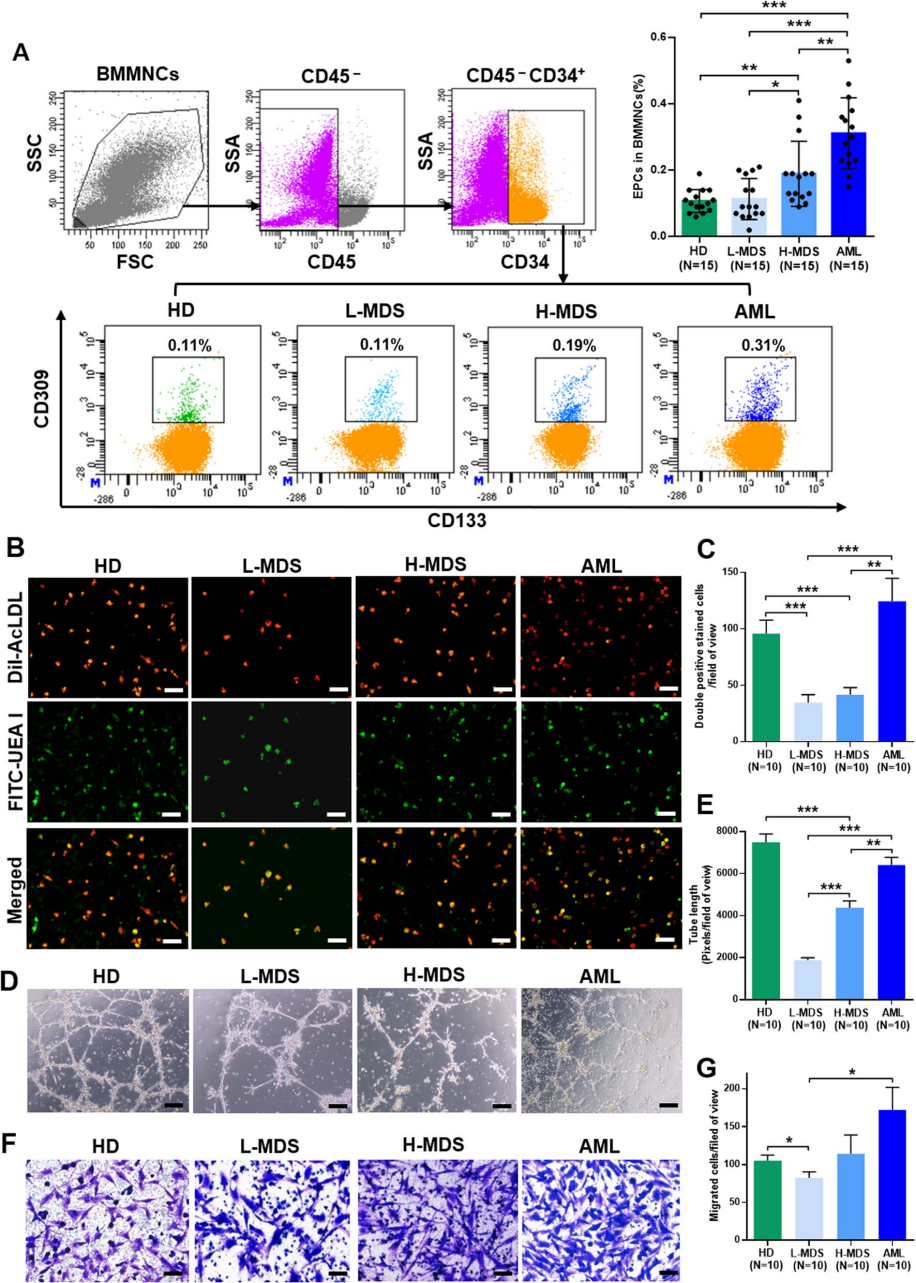
The number and functions of BM EPCs from patients with MDS. HD and patients with de novo AML were enrolled as controls. **A, left** The EPC phenotype was characterized by demonstrating positive expression of CD34, CD309 and CD133 by flow cytometry. The **A, right** percentage of BM EPCs in precultivated BMMNCs was analysed. **B** Representative images (scale bars represent 200 µm) of typical cultured BM EPCs collected at day 7 of culture from HDs and L-MDS, H-MDS and AML patients were characterized by double-positive staining (merged in yellow) with DiI-AcLDL (red) and FITC-UEA I (green) (original magnification, 10×). **C** Quantification of double-positive EPCs/field of view (merged in yellow) stained with DiI-AcLDL (red) and FITC-UEA I (green) at day 7 of culture (original magnification, 10×). **D** Representative images (scale bars represent 200 µm) of tube formation (pixels of tubes per field of view) by BM EPCs at day 7 of culture (original magnification, 10×). **E** Quantification of tube length (pixels of tubes per field of view) of BM EPCs at day 7 of culture (original magnification, 10×). **F** BM EPCs at day 7 of culture were cultured in a transwell chamber for 24 h, fixed and then stained with crystal violet. Representative images (scale bars represent 200 µm) of migrated cells on the bottom surface of the membrane (original magnification, 10×). **G** The number of migrated BM EPCs per field of view was compared (original magnification, 10×). Three power fields were randomly counted and averaged per sample. Statistical analyses were performed using the Mann–Whitney U test. Data are presented as the means ± SEM (*P ≤ 0.05, **P ≤ 0.005, ***P ≤ 0.001). *AML* Acute myeloid leukaemia, *BM* Bone marrow, *BMMNCs* Bone marrow mononuclear cells, *EPCs* Endothelial progenitor cells, *DiI-Ac-LDL* DiI-acetylated low-density lipoprotein, *FITC-UEA I* FITC-labelled Ulex Europaeus Agglutinin I, *HD* Healthy donor, *H-MDS* Higher-risk myelodysplastic syndromes, *L-MDS* Lower-risk myelodysplastic syndromes

The representative BM EPC phenotype was characterized by CD34^+^CD309^+^CD133^+^ by flow cytometry (Fig. 1A). The percentage of BM EPCs from higher-risk MDS patients (higher-risk MDS BM EPCs) (Fig. 1A, 0.19% ± 0.03% vs. 0.11% ± 0.02%, *P* = 0.03) was significantly higher than BM EPCs from lower-risk MDS patients (lower-risk MDS BM EPCs), whereas the percentage of primary BM EPCs from AML patients (AML BM EPCs) was higher than higher-risk MDS BM EPCs (Fig. 1A, 0.31% ± 0.03% vs. 0.19% ± 0.03%, *P* = 0.002).

After 7 days of cultivation, spindle-shaped and elongated BM adherent cells were further functionally characterized as EPCs, which were capable of DiI-AcLDL uptake and FITC-UEA I binding (the typical functional EPC markers) [28–30, 40, 41]. Moreover, EPC identity was confirmed by qRT-PCR for endothelial specific marker genes (VEGFR2, VE-cadherin and VWF) in Additional file 1: Fig. S1. The number of double-positive stained AML BM EPCs was significantly higher than higher-risk MDS BM EPCs (Fig. 1B and C, 124.5 ± 20.3 vs. 41.7 ± 6.3, *P* = 0.003). Together, increased numbers of BM EPCs were found in MDS patients, especially in more severe types of MDS.

### Decreased angiogenic potential but increased migration ability of BM EPCs in MDS patients

To evaluate the angiogenic potential and migration of BM EPCs from different types of MDS patients, tube formation and migration were analysed on day 7 of culture [28–30, 40]. Higher-risk MDS BM EPCs showed a significantly increased tube formation ability compared with lower-risk MDS BM EPCs (Fig. 1D and E, 4375 ± 321 vs.1874.7 ± 118.9, *P* < 0.0001) and a markedly decreased tube formation ability compared with AML patients (Fig. 1D and E, 4375 ± 321 vs. 6403 ± 370.4, *P* = 0.001). The AML BM EPCs on day 7 of culture showed increased migrated cells (Fig. 1F and G, 172.4 ± 29.8 vs. 82.6 ± 7.9, *P* = 0.01) compared with lower-risk MDS BM EPCs. These results demonstrated that the decreased angiogenic potential but increased migration capability in BM EPCs from MDS patients.

### RNA-seq indicates the progressive change and differences of haematopoiesis- and immune-related pathways and genes in MDS BM EPCs

**Fig. 2:**
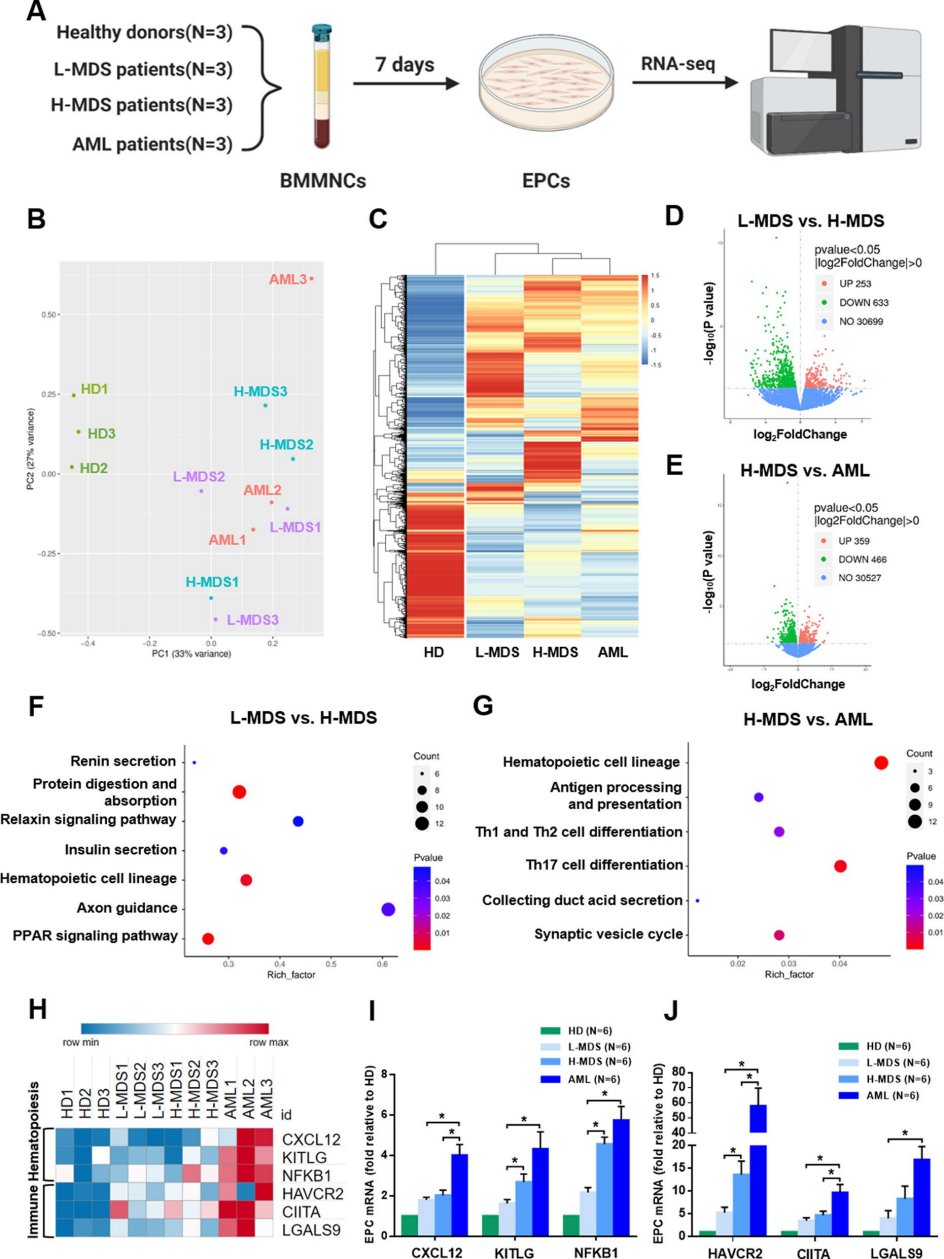
Transcriptome heterogeneity of BM EPCs from MDS patients identified by bulk RNA-seq. **A** Schematic of the experiment. BM EPCs from 3 HDs and 3 L-MDS, 3 H-MDS, and 3 AML patients at 7 days of culture were collected, and bulk RNA-seq was performed. **B** PCA score plot of 12 libraries. **C** Heatmap and hierarchical clustering of RNA-seq data for the HD, L-MDS, H-MDS and AML groups using the Euclidean distance. Distributions and quantifications of genes in BM EPCs from **D** L-MDS verses H-MDS and **E** H-MDS verses AML. The x axis shows the log2 of gene expression change, whereas the y axis shows the − log10 of the *P* value. The KEGG pathway enrichment analyses of Organismal Systems class of the different genes between **F** L-MDS vs. H-MDS and **G** H-MDS vs. AML are shown. The x axis shows the rich factor. **H** Heatmap shows haematopoiesis- and immune-related gene expression in bulk RNA-seq data of 12 libraries. The relative mRNA levels of **I** *CXCL12*, *KITLG*, *NFKB1* genes and **J** *HAVCR2*, *CIITA*, *LGALS9* genes in HD, L-MDS, H-MDS and AML BM EPCs were determined using qRT-PCR. The relative mRNA analyses were using Wilcoxon matched-pairs signed rank test. Data are presented as the means ± SEM (*P ≤ 0.05). *AML* Acute myeloid leukaemia, *BM* Bone marrow, *BMMNCs* Bone marrow mononuclear cells, *EPCs* Endothelial progenitor cells, *HD* Healthy donor, *H-MDS* Higher-risk myelodysplastic syndromes, *KEGG* Kyoto Encyclopedia of Genes and Genomes, *L-MDS* Lower-risk myelodysplastic syndromes, *PCA* Principal component analysis, *RNA-seq* RNA sequencing, *ROS* reactive oxygen species

To uncover the underlying mechanism of the variant dysfunctions in different types MDS BM EPCs, the lower-risk MDS BM EPCs (N = 3), higher-risk MDS BM EPCs (N = 3), AML patients BM EPCs (N = 3) and primary BM EPCs from HDs (HD BM EPCs) (N = 3) were analysed via RNA-seq (Fig. 2A). The results of PCA (Fig. 2B) and hierarchical clustering analysis (Fig. 2C) showed that the entire population was clearly separated into two distinct subpopulations, HD and disease BM EPCs. More importantly, the heatmap (Fig. 2C) of different groups showed the progressive change in the total RNA expression profile of HD BM EPCs, lower-risk MDS BM EPCs, and higher-risk MDS BM EPCs to AML BM EPCs. There were 886 different genes between lower-risk MDS and higher-risk MDS BM EPCs (Fig. 2D) and 825 different genes between higher-risk MDS and AML BM EPCs (Fig. 2E). KEGG pathway enrichment analysis of Organismal Systems class (Fig. 2F and G) indicated that haematopoiesis- and immune-related pathways were enriched in MDS BM EPCs. For example, haematopoietic cell lineage was enriched in both lower-risk MDS BM EPCs vs. higher-risk MDS BM EPCs and higher-risk MDS BM EPCs vs. AML BM EPCs. Th17 and Th2 cell differentiation and Th17 cell differentiation were enriched in higher-risk MDS BM EPCs vs. AML BM EPCs.

In addition, the previously reported haematopoiesis- and immunomodulation-related genes [51–56] in BM EPCs were detected by RNA-seq (Fig. 2H) and verified by qRT-PCR. As shown in Fig. 2H–J, mRNA levels of haematopoiesis-related genes *CXCL12*, *KITLG*, *NFKB1* and immune-related genes *HAVCR2*, *LGALS9*, *CIITA* were increased in higher-risk MDS and AML BM EPCs compared with lower-risk MDS BM EPCs. These results suggested markedly enriched haematopoiesis- and immunomodulatory-related pathways and upregulation of related genes in higher-risk MDS BM EPCs. However, normal or malignant haematopoiesis-related genes could not be distinguished via RNA-seq analysis. Therefore, we further carried out in vitro experiments to analyse the haematopoiesis and immunomodulatory function of BM EPCs from MDS patients.

### Decreased ability of BM EPCs to support HSCs in higher-risk MDS patients

**Fig. 3:**
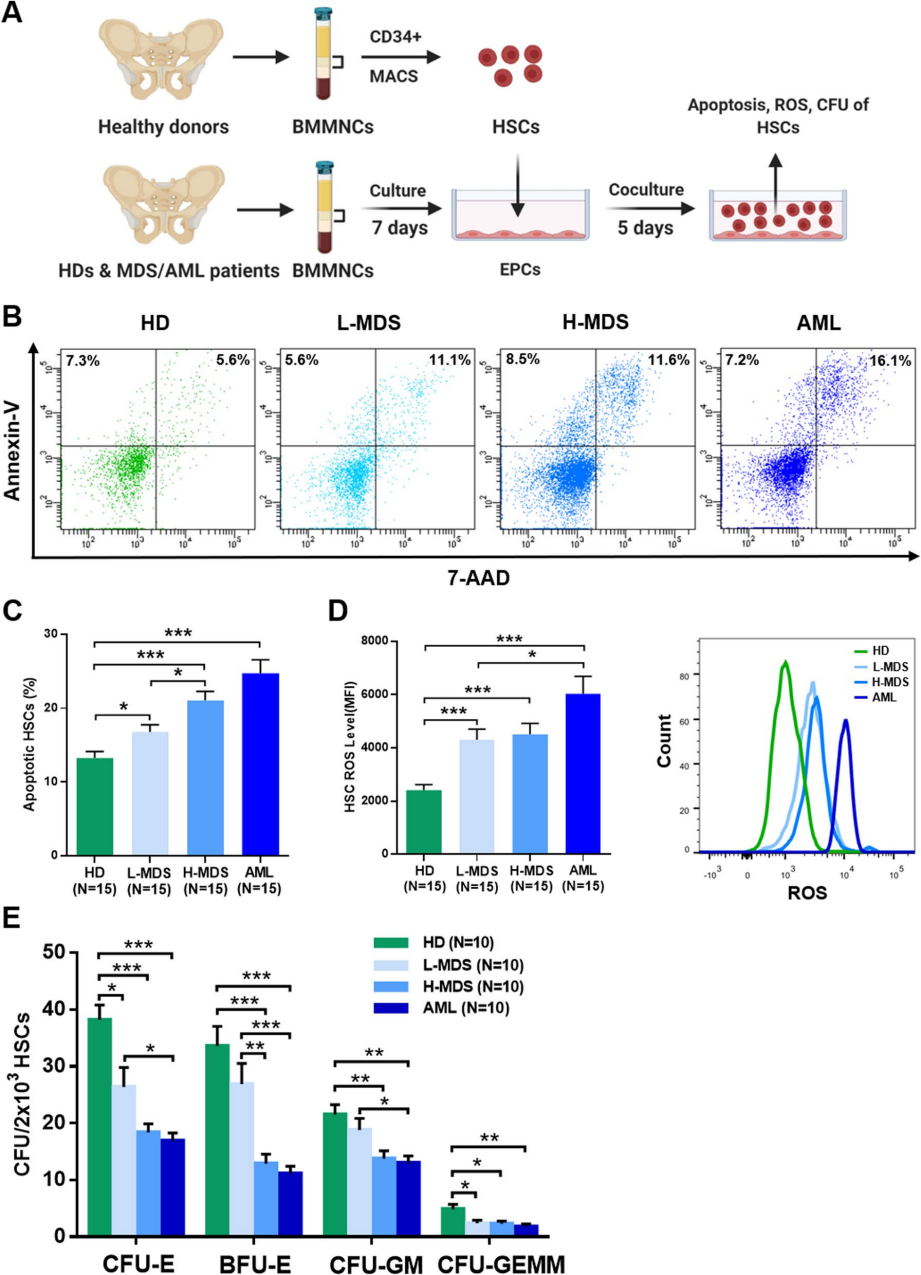
Supporting abilities of BM EPCs from MDS patients to HSCs. **A** Schematic diagram of the study design for the BM EPC coculture process with HSCs. After 5 days of coculture, the apoptosis ratio and the quantification of the level of intracellular ROS and CFU efficiencies of HSCs were detected. Representative images (**B**) and quantification (**C**) of the apoptosis ratio of HSCs after coculture with BM EPCs are shown. Quantification (**D**, left) and representative images (**D**, right) of the ROS levels (MFI) of HSCs after coculture are shown. **E** The CFU plating efficiencies of HSCs, including CFU-E, BFU-E, CFU-GM and CFU-GEMM, after coculture with BM EPCs from HDs, L-MDS, H-MDS and AML patients. Statistical analyses were performed using the Mann–Whitney U test. Data are presented as the means ± SEM (*P ≤ 0.05, ** *P* ≤ 0.005, *** *P* ≤ 0.001). *AML* Acute myeloid leukaemia, *BFU-E* Burst-forming unit erythroid, *BM* Bone marrow, *BMMNCs* Bone marrow mononuclear cells, *CFU* Colony-forming unit, *CFU-E* Colony-forming unit erythroid, *CFU-GM* Colony-forming unit-granulocyte/macrophages, *CFU-GEMM* Colony-forming unit-granulocyte, erythroid, macrophage and megakaryocyte, *EPCs* Endothelial progenitor cells, *HD* Healthy donor, *HSC* haematopoietic stem cells, *H-MDS* Higher-risk myelodysplastic syndromes, *L-MDS* Lower-risk myelodysplastic syndromes

To investigate the effects of BM EPCs on HSCs in vitro, we sorted CD34^+^ cells from BMMNCs of HDs with magnetic beads and cocultured them with BM EPCs. After 5 days, we analysed the apoptosis rates, intracellular ROS levels and CFU plating efficiencies of HSCs (Fig. 3A). Compared with the lower-risk MDS group, the higher-risk MDS group exhibited a marked increase in the apoptosis rates of HSCs after coculture (Fig. 3B and C, 21.09 ± 1.21% vs. 16.79 ± 0.97%, *P* = 0.01). The ROS level (Fig. 3D, 6039 ± 654.4 vs. 4308 ± 394.7, *P* = 0.03) of CD34^+^ cells after coculture with AML BM EPCs was significantly higher than that of lower-risk MDS BM EPCs. HSCs cocultured with higher-risk MDS BM EPCs had lower CFU plating efficiencies than those cocultured with lower-risk MDS BM EPCs (Fig. 3E), as determined by CFU-E, BFU-E (12.9 ± 1.67 vs. 26.9 ± 3.62, *P* = 0.003), CFU-GM, and CFU-GEMM. The AML group had lower CFU-E, BFU-E, CFU-GM and CFU-GEMM plating efficiencies than the higher-risk MDS group. These data suggest that of MDS BM EPCs were less able to support HSCs in more severe type of MDS patients.

### BM EPCs induce T cell differentiation towards more immune-tolerant cells in higher-risk MDS patients

**Fig. 4:**
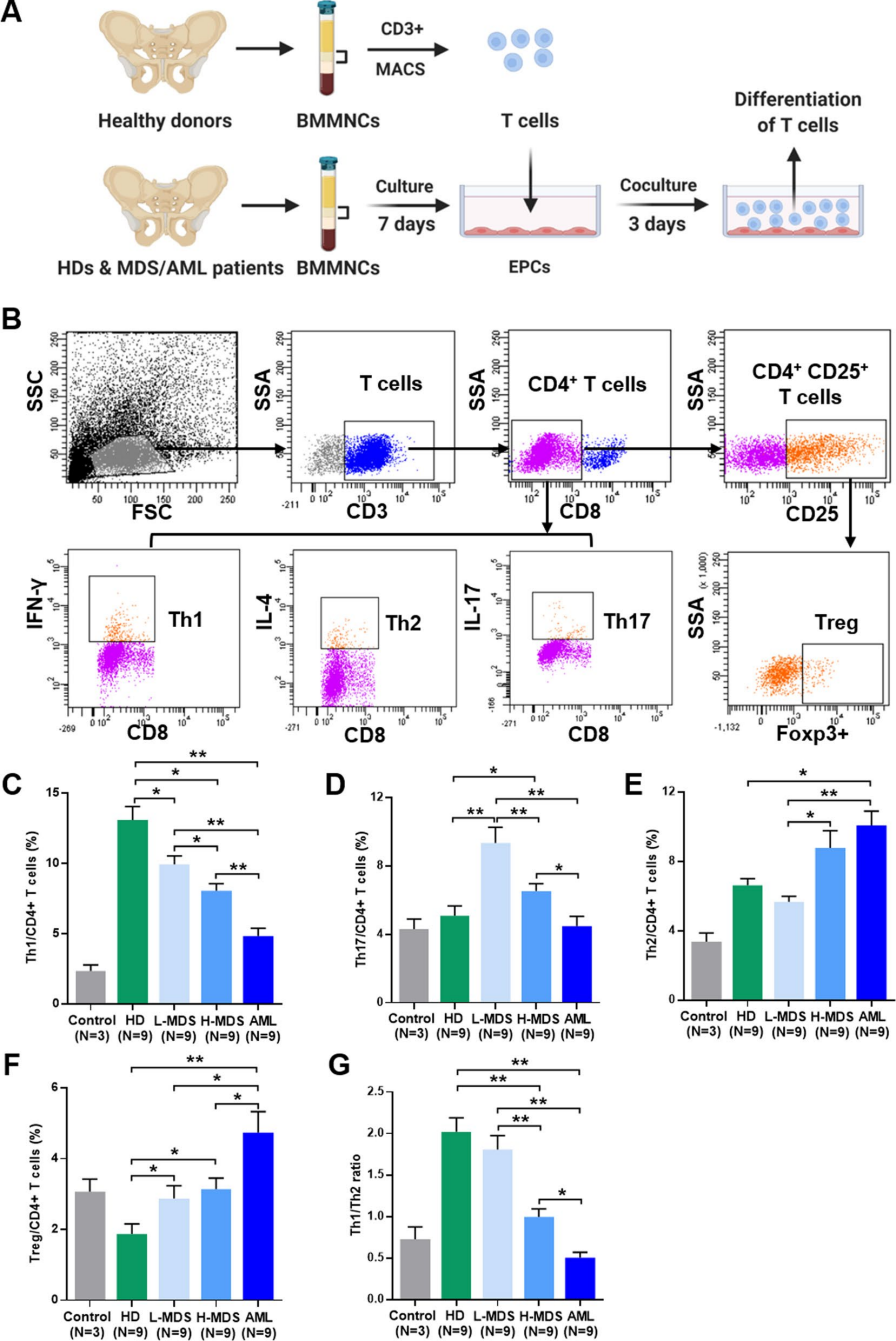
Regulatory abilities of BM EPCs from MDS patients to differentiate T cells. **A** Schematic diagram of the study design for BM EPC coculture processes with T cells. After 3 days of coculture, the differentiation of T cells was analysed by flow cytometry. **B** The subtypes of Th1 cells, Th2 cells, Th17 cells and Tregs were characterized by positive expression of CD4^+^IFN-γ^+^, CD4^+^IL-4^+^, CD4^+^IL-17^+^ and CD4^+^CD25^+^Foxp3^+^, respectively. The percentage of **C** Th1 cells, **D** Th17 cells, **E** Th2 cells, **F** Tregs and **G** the ratio of Th1/Th2 cells after coculture without (Control) or with HD-derived, L-MDS, H-MDS, AML patient-derived BM EPCs for 3 days. The paired analyses were using Wilcoxon matched-pairs signed rank test. Data are presented as the means ± SEM (*P ≤ 0.05, **P ≤ 0.005). *AML* Acute myeloid leukaemia, *BM* Bone marrow, *BMMNCs* Bone marrow mononuclear cells, *EPCs* Endothelial progenitor cells, *HD* Healthy donor, *H-MDS* Higher-risk myelodysplastic syndromes, *L-MDS* Lower-risk myelodysplastic syndromes, *Th* T helper, *Tregs* Regulatory T cells, *ROS* reactive oxygen species

To further investigate the immunoregulatory effects of BM EPCs in vitro, we sorted CD3^+^ cells from BMMNCs of HDs and cocultured them without or with HD BM EPCs, lower-risk MDS BM EPCs, higher-risk MDS BM EPCs and AML BM EPCs. After 3 days, we analysed differences in T cell subsets after coculture or not (control of T cell alone cultures) (Fig. 4A). The subtypes of Th1 cells, Th2 cells, Th17 cells and Tregs were characterized by positive expression of CD4^+^IFN-γ^+^, CD4^+^IL-4^+^, CD4^+^IL-17^+^ and CD4^+^CD25^+^Foxp3^+^, respectively (Fig. 4B). There was a markedly lower percentage of Th1 cells in the AML group than in the higher-risk MDS group (Fig. 4C, 4.86% ± 0.54% vs. 8.07% ± 0.51%, *P* = 0.004) and in the higher-risk MDS group than in the lower-risk MDS group (Fig. 4C, 8.07% ± 0.51% vs. 9.95% ± 0.6%, *P* = 0.04). A significantly lower percentage of Th17 cells was observed in the higher-risk MDS group than in the lower-risk MDS group (Fig. 4D, 6.54% ± 0.43% vs. 9.35% ± 0.92%, *P* = 0.004) and in the AML group than in the higher-risk MDS group (Fig. 4D, 4.48% ± 0.58% vs. 6.54% ± 0.43%, *P* = 0.04). However, higher percentages of Th2 cells (Fig. 4E, 8.77% ± 1.0% vs. 5.67% ± 0.31%, *P* = 0.02) in the higher-risk MDS group than in the lower-risk MDS group. In addition, higher percentages of Tregs (Fig. 4F, 4.74% ± 0.59% vs. 3.14% ± 0.31%, *P* = 0.01) were observed in the AML group than in the higher-risk MDS group. As a result, the ratio of Th1/Th2 cells was markedly lower in the higher-risk MDS group than in the lower-risk MDS group (Fig. 4G, 1.0 ± 0.1 vs. 1.81 ± 0.17, *P* = 0.004), whereas that was significantly lower in the AML group than in the higher-risk MDS group (Fig. 4G, 0.51 ± 0.06 vs. 1.0 ± 0.1, *P* = 0.02). These data showed that lower-risk MDS BM EPCs regulated T cell differentiation into Th17 cells, whereas higher-risk MDS and AML BM EPCs regulated T cell differentiation into Th2 cells and Tregs. Our data suggested that BM EPCs might be inclined to induce T cell differentiation towards more immune-tolerant cells in higher-risk MDS patients.

### Increased ability of BM EPCs to support leukaemia cells in higher-risk MDS patients

**Fig. 5:**
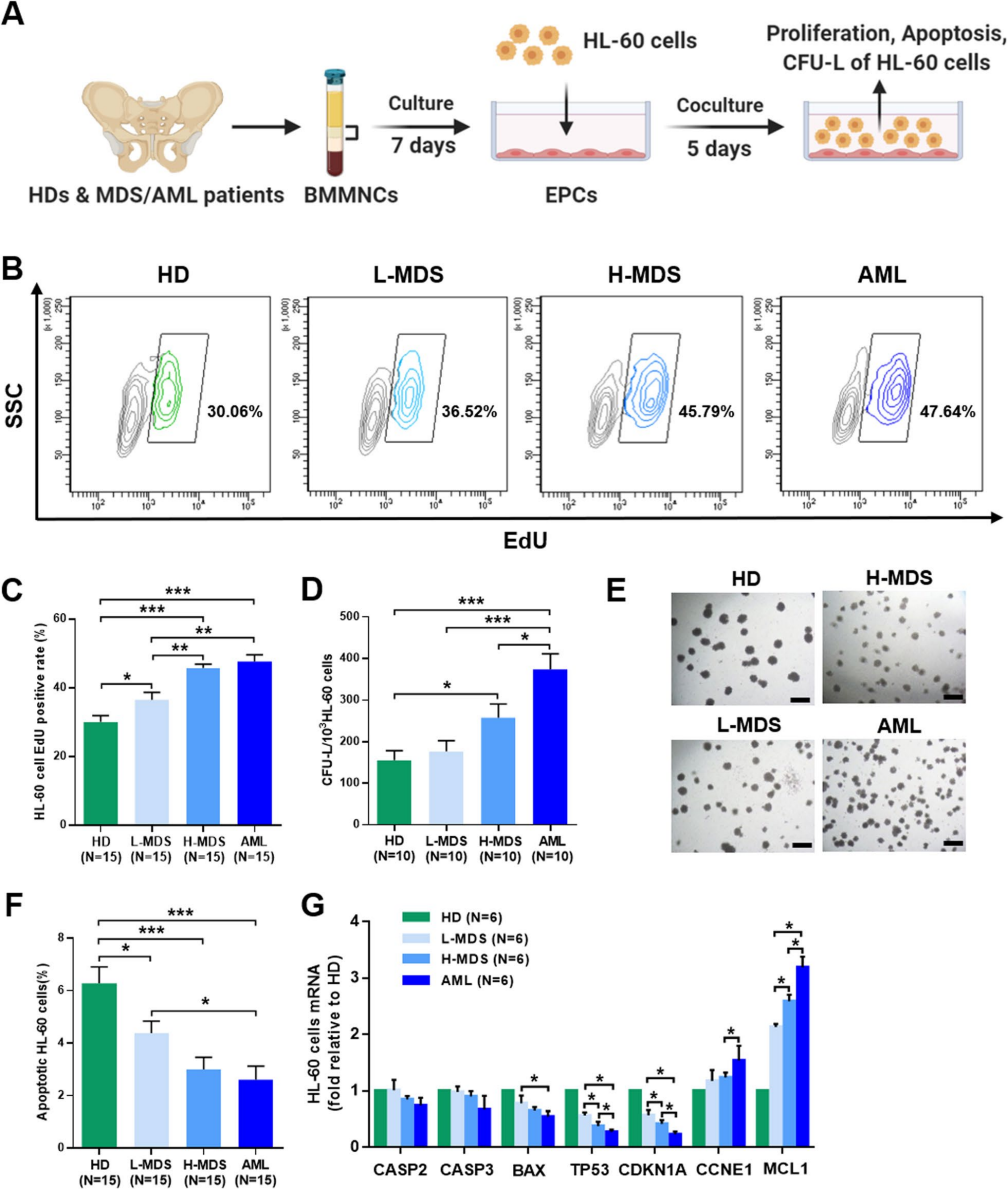
Supporting abilities of BM EPCs from MDS patients to leukaemia cells. **A** Schematic diagram of the study design for BM EPC coculture processes with HL-60 cells. After 5 days of coculture, the EdU-positive rates, apoptosis ratio and CFU-L efficiencies of HL-60 cells were detected. Representative images (**B**) and quantification of the EdU-positive rates (**C**) of HL-60 cells after coculture with BM EPCs are shown. Quantification (**D**) of the CFU-L plating efficiencies and representative images (scale bars represent 500 μm) (**E**) of CFU-L after coculture with BM EPCs are shown (original magnification, 4×). Three power fields were randomly counted and averaged per sample. **F** Quantification of the apoptosis ratio of HL-60 cells after coculture. **G** The relative mRNA levels of *CASP2, CASP3, BAX, TP53, CDKN1A*, *CCNE1* and *MCL1* in HL-60 cells after coculture with BM EPCs were determined by qRT‐PCR. Statistical analyses were performed using the Mann–Whitney U test. The relative mRNA analyses were using Wilcoxon matched-pairs signed rank test. Data are presented as the means ± SEM (*P ≤ 0.05, **P ≤ 0.005, ***P ≤ 0.001). *AML* Acute myeloid leukaemia, *BM* Bone marrow, *BMMNCs* Bone marrow mononuclear cells, *CFU-L* Leukaemia colony-forming unit, *EdU* 5-ethynyl-20 deoxyuridine, *EPCs* Endothelial progenitor cells, *HD* Healthy donor, *H-MDS* Higher-risk myelodysplastic syndromes, *L-MDS* Lower-risk myelodysplastic syndromes

To investigate the effect of BM EPCs on leukaemia cells in vitro, we assessed the proliferation, apoptosis and CFU-L plating efficiency of HL-60 cells after coculture with BM EPCs (Fig. 5A). In addition, the relative mRNA expression levels of apoptosis- and cell cycle-related genes were detected in HL-60 cells after coculture. The EdU -positive rate of HL-60 cells was significantly higher in the higher-risk MDS group than in the lower-risk MDS group (Fig. 5B and C, 45.79% ± 1.12% vs. 36.52% ± 2.20%, *P* = 0.003). More importantly, CFU-L plating efficiencies were markedly increased in AML group compared with higher-risk MDS group (Fig. 5D and E, 374.1 ± 37.2 vs. 257.6 ± 33.2, *P* = 0.04). However, the apoptotic rate of HL-60 cells was notably decreased in the AML group compared with the lower-risk MDS group (Fig. 5F, 2.6% ± 0.52% vs. 4.38% ± 0.46%, *P* = 0.01). The *CASP2*, *CASP3*, *BAX*, *TP53* and *CDKN1A* mRNA levels in HL-60 cells were downregulated, whereas the *CCNE1* and *MCL1* mRNA levels were upregulated in the higher-risk MDS and AML groups than the lower-risk MDS group (Fig. 5G). These data suggest that BM EPCs may become more supportive of leukaemia cells in higher-risk MDS patients.

### The levels of ROS and the apoptosis ratio were increased in BM EPCs from higher-risk MDS patients

**Fig. 6:**
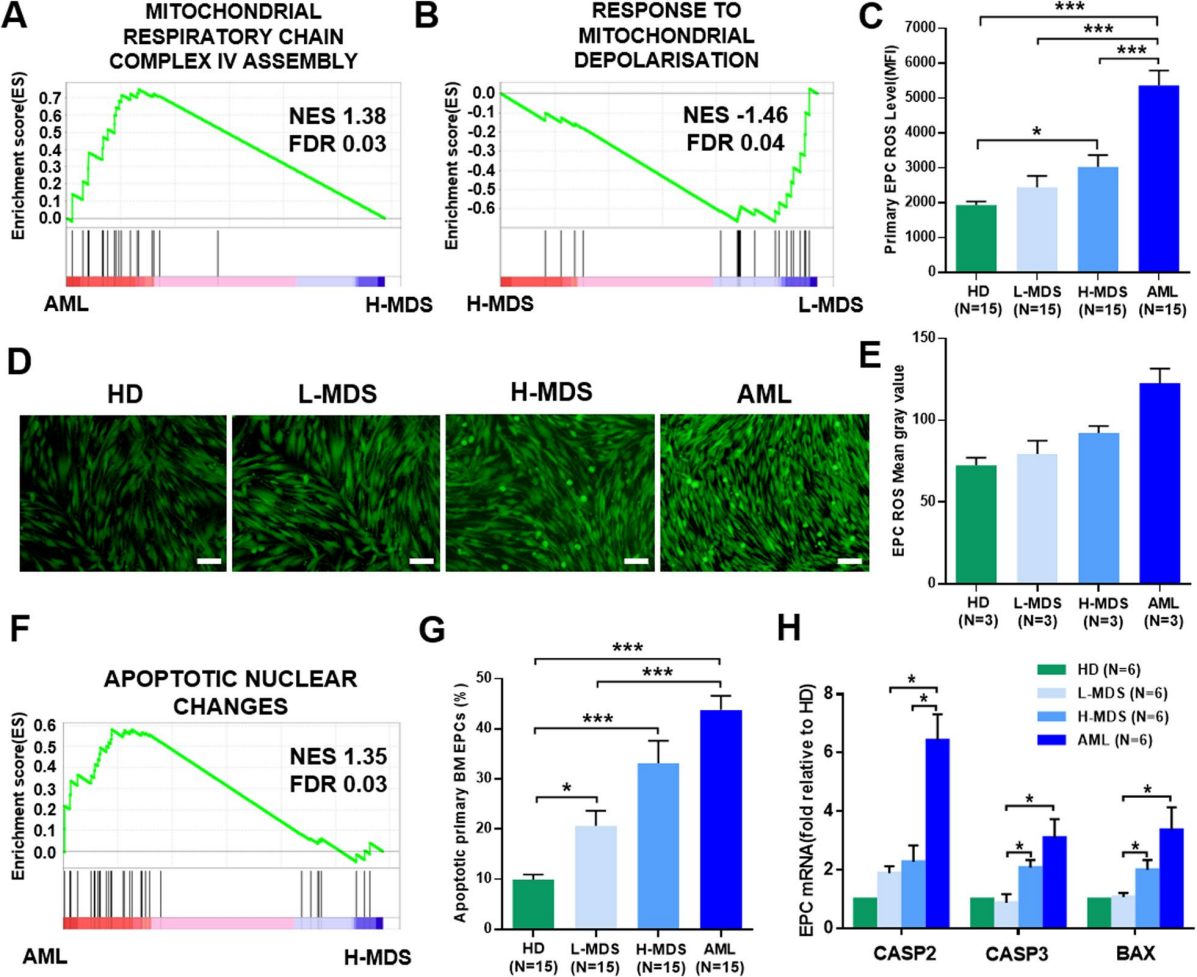
The levels of ROS and apoptosis ratio in BM EPCs from MDS patients. **A** and **B** GSEA was performed with differentially expressed genes in AML BM EPCs versus H-MDS BM EPCs or H-MDS BM EPCs versus L-MDS BM EPCs, highlighting enrichment in mitochondria-related signaling pathways. **C** ROS levels of precultivated BM EPCs from HD, L-MDS, H-MDS and AML patients. Representative images (**D**) (scale bars represent 200 μm) of the BM EPCs at day 7 in culture after incubation with 2ʹ,7ʹ-dichlorofluorescence diacetate (original magnification, 10×). **E** Quantification of the mean ROS grey value of the BM EPCs at day 7 in culture after incubation with 2ʹ,7ʹ-dichlorofluorescence diacetate (original magnification, 10×). Three power fields were randomly counted and averaged per sample. The mean grey value of ROS was improved in AML compared with H-MDS and increased in H-MDS compared with L-MDS, but the results were not statistically significant. **F** GSEA was performed with differentially expressed genes in AML BM EPCs versus H-MDS BM EPCs, highlighting the enrichment in the apoptosis signaling pathway. **G** The apoptosis ratio of precultivated BM EPCs from HDs, L-MDS, H-MDS and AML patients. **H** The relative mRNA levels of *CASP2*, *CASP3* and *BAX* in BM EPCs at day 7 in culture from HDs, L-MDS, H-MDS and AML patients were determined using qRT-PCR. Statistical analyses were performed using Mann–Whitney U test. The relative mRNA analyses were using Wilcoxon matched-pairs signed rank test. Data are presented as the means ± SEM (*P ≤ 0.05, **P ≤ 0.005, ***P ≤ 0.001). *AML* Acute myeloid leukaemia, *BM* Bone marrow, *EPCs* Endothelial progenitor cells, *GSEA* Gene set enrichment analysis, *HD* Healthy donor, *H-MDS* Higher-risk myelodysplastic syndromes, *L-MDS* Lower-risk myelodysplastic syndromes, *ROS* reactive oxygen species

**Fig. 7:**
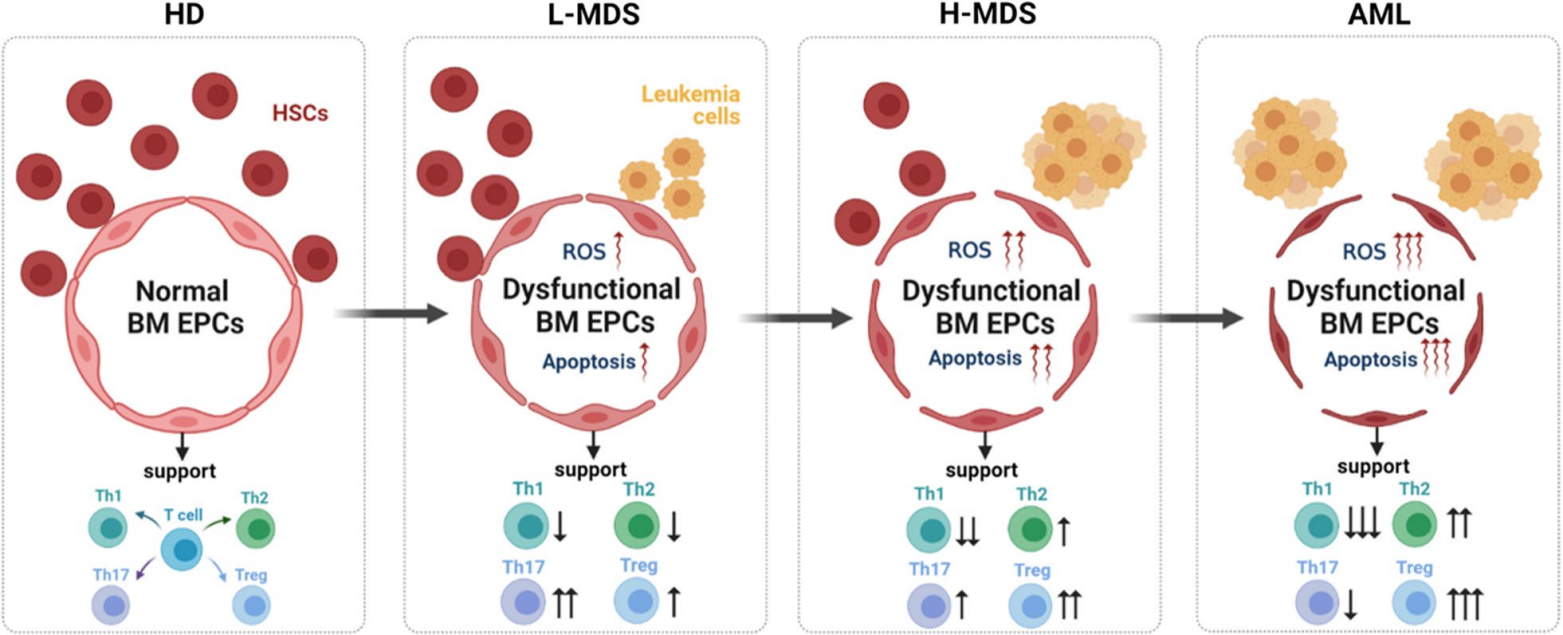
Graphical abstract of the current study. Schematic illustration of the dysfunctional BM EPCs in HDs, patients with lower-risk MDS, higher-risk MDS and AML. The abilities of BM EPCs from higher-risk MDS patients to support HSCs decreased, whereas to support leukaemia cells increased. Furthermore, BM EPCs might be inclined to induce T cell differentiation towards more immune-tolerant cells in higher-risk MDS patients. In addition, excessive production of ROS and apoptotic pathway activation may be the underlying mechanisms of dysfunctional BM EPCs. *AML* Acute myeloid leukaemia, *BM* Bone marrow, *EPCs* Endothelial progenitor cells, *HD* Healthy donor, *H-MDS* Higher-risk myelodysplastic syndromes, *HSC* Haematopoietic stem cell, *L-MDS* Lower-risk myelodysplastic syndromes, *Th* T helper, *Tregs* T cells, *ROS* reactive oxygen species

To further explore the internal changes in dysfunctional EPCs and identify underlying therapeutic targets, we investigated damage related pathways from the RNA-seq data. GSEA highlighted abnormalities of mitochondrial signaling pathway (Fig. 6A, B), so we verified them by detecting ROS levels of precultivated and cultivated BM EPCs. The ROS level of precultivated AML BM EPCs was significantly higher than that in higher-risk MDS BM EPCs (Fig. 6C, 5369 ± 426.0 vs. 3039 ± 335.3, *P* = 0.0002). The ROS level in the cultivated AML BM EPCs was higher than that in the cultivated higher-risk MDS BM EPCs, whereas the ROS level of the cultivated higher-risk MDS BM EPCs was higher than that in the lower-risk MDS BM EPCs (Fig. 6D, E). Moreover, the pathway of apoptotic nuclear changes was identified in GSEA (Fig. 6F). The apoptosis rate (Fig. 6G, 43.79% ± 2.77% vs. 20.63% ± 3.01%, *P* < 0.0001) was significantly higher in AML BM EPCs than in lower-risk MDS EPCs. The relative mRNA expression levels of apoptosis-related genes in BM EPCs were further analysed by qRT-PCR. The *CASP2* mRNA level was significantly upregulated in AML BM EPCs (Fig. 6H, 6.43 ± 0.89-fold vs. 2.28 ± 0.56-fold, *P* = 0.03) compared to higher-risk MDS BM EPCs. The *CASP3* (Fig. 6H, 2.09 ± 0.26-fold vs. 0.88 ± 0.3-fold, *P* = 0.03) and *BAX* (Fig. 6H, 2.0 ± 0.35-fold vs. 1.08 ± 0.14-fold, *P* = 0.03) mRNA levels were significantly upregulated in higher-risk MDS BM EPCs compared to lower-risk MDS BM EPCs. Together, elevated levels of ROS and apoptosis of BM EPCs were found in MDS patients, especially in higher-risk MDS patients, which may be the underlying repair targets for MDS patients (Fig. 7).

## Discussion

The current study firstly demonstrated increased but dysfunctional BM EPCs in patients with MDS. The abilities of BM EPCs from higher-risk MDS patients to support HSCs decreased, whereas those from higher-risk MDS patients to support leukaemia cells increased. Furthermore, BM EPCs might be inclined to induce T cell differentiation towards more immune-tolerant cells in more severe type of MDS patients. In addition, excessive production of ROS and apoptotic pathway activation may be the underlying mechanisms of dysfunctional BM EPCs. Our data indicate that repair of dysfunctional BM EPCs may be a potential therapeutic approach for MDS patients.

Increased BM angiogenesis has been reported in MDS patients [32, 57–59]. Immunohistochemical studies have demonstrated an increased microvessel density (MVD) in the biopsies of MDS patients [58–60]. The results concerning the correlation between BM MVD and classification are controversial. Some studies reported a higher MVD preferentially among higher-risk MDS patients [58, 61], while other studies failed to find such a correlation [60]. Other studies regarding circulating endothelial cells and peripheral EPCs are consistently increased in MDS patients compared with normal controls [32, 57]. In summary, the number of BM EPCs in MDS patients and their correlation with the different risk degrees are not clear. More importantly, the functions of BM EPCs in patients with different risk groups of MDS are largely unknown. Our study firstly demonstrated increased BM EPCs with dysfunctions in MDS patients, especially more severe dysfunctions of BM EPCs in patients with higher-risk MDS. We speculated that the increased and dysfunctional EPCs in MDS patients were responsible for more nutrient and metabolite turnover, and may secrete more cytokines to better support malignant hematopoiesis. Based on our previous work and the current study, the impairment of EPCs was associated with elevated intracellular ROS levels and an elevated apoptosis ratio in MDS patients [28, 29, 39, 62]. Therefore, it is worth investigating the efficacy of therapeutic strategies, such as *N*-acetyl-l-cysteine (a reactive oxygen species scavenger) [28, 29, 62, 63] or inhibitor of EPC-targeted apoptosis pathways, to improve the prognosis of MDS patients by enhancing BM EPCs in the future.

A great deal of researches have certified the critical role of EPCs in regulating haematopoiesis [24, 26, 27], whereas Hatfield et al. found that EPCs supported leukaemia cells by directly enhancing the proliferation and inhibiting apoptosis of AML blasts [45]. However, it is unclear whether the abilities of BM EPCs from MDS patients to support HSCs or leukaemia cells are the same or different. Surprisingly, we found antipodal results for normal HSCs and leukaemia cells. The CFU plating efficiency of HSCs declined with disease progression, which suggested BM EPCs from patients with MDS or AML could not support normal differentiation of HSCs. On the contrary, the CFU-L plating efficiency increased, which implied the possible role of BM EPCs as an oncogenic driver or facilitator of MDS. On the other hand, the progression of MDS is also facilitated by immune deregulation [1]. However, the correlation between BM EPCs and immune deregulation in MDS is largely unknown. Consistent with previous clinical reports of T cell subtypes in MDS patients [21, 22], our data demonstrated that BM EPCs from lower-risk MDS patients regulate T cell differentiation into inflammatory Th17 cells but BM EPCs from higher-risk MDS patients regulate T cells into more immune tolerant cells.

We are aware that the underlying mechanism on how BM EPCs regulate T cells and the precise T cell subset need to be further explored in the future. However, our data indicated that the dysregulated immunomodulatory function of EPCs may also contribute to ineffective haematopoiesis and evasion from antitumoural immunity in MDS. Therefore, in conjunction with existing therapies, improvement of BM EPCs may be a potential therapeutic strategy for patients with MDS.

## Conclusions

In summary, the current study demonstrated that dysfunctional BM EPCs were involved in MDS patients. Changes in the BM microenvironment may be a primary driver of human haematological malignancies, as has been suggested by animal models [64, 65]. Although further validation is required, our findings indicate that improving BM EPCs may represent a potential therapeutic approach for MDS patients.

## Supplementary Information

**Additional file 1: Figure S1.** Validation of EPC identity. **Table S1.** Clinical characteristics of MDS patients. **Table S2.** Antibody information. **Table S3.** Top 20 up and down regulated genes in higher-risk MDS BM EPCs than lower-risk MDS BM EPCs.** Table S4.** Top 20 up and down regulated genes in AML BM EPCs than higher-risk MDS BM EPCs. **Table S5.** The primer sequences of genes used for qRT-PCR.

## Data Availability

All data needed to evaluate the conclusions in the paper are present in the paper and/or the Additional file. The accession number of whole transcriptome RNA-seq data is GSE197907.

## Abbreviations

Allo-HSCT: Allogeneic haematopoietic stem cell transplantation
AML: Acute myeloid leukaemia
BFU-E: Burst-forming unit erythroid
BM: Bone marrow
BMMNCs: Bone marrow mononuclear cells
CFU: Colony-forming unit
CFU-E: Colony-forming unit erythroid
CFU-GM: Colony-forming unit-granulocyte/macrophages
CFU-GEMM: Colony-forming unit-granulocyte, erythroid, macrophage and megakaryocyte
CFU-L: Leukaemia colony-forming unit
DEG: Differential gene expression
DiI-Ac-LDL: DiI-acetylated low-density lipoprotein
EdU: 5-Ethynyl-20-deoxyuridine
EPCs: Endothelial progenitor cells
FITC-UEAI: FITC-labelled Ulex Europaeus Agglutinin I
GSEA: Gene set enrichment analysis
HDs: Healthy donors
HSC: Haematopoietic stem cell
IPSS-R: Revised International Prognostic Scoring System
KEGG: Kyoto Encyclopedia of Genes and Genomes
MDS: Myelodysplastic syndromes
PCA: Principal component analysis
qRT-PCR: Real-time quantitative polymerase chain reaction
RNA-seq: RNA sequencing
ROS: Reactive oxygen species
Th: T helper
Tregs: T cells
VEGFR2: Vascular endothelial growth factor receptor 2
7-AAD: 7-Amino-actinomycin D
