# Supplementary material for: Dysfunctional bone marrow endothelial progenitor cells are involved in patients with myelodysplastic syndromes
Source: J Transl Med. 2022 Mar 29;20:144. doi: 10.1186/s12967-022-03354-2 (PMC8962499; doi:10.1186/s12967-022-03354-2)
Supplement: Supplementary file 1 — Additional file 1: Figure S1. Validation of EPC identity. Table S1. Clinical characteristics of MDS patients. Table S2. Antibody information. Table S3. Top 20 up and down regulated genes in higher-risk MDS BM EPCs than lower-risk MDS BM EPCs. Table S4. Top 20 up and down regulated genes in AML BM EPCs than higher-risk MDS BM EPCs. Table S5. The primer sequences of genes used for qRT-PCR. [file 12967_2022_3354_MOESM1_ESM.docx]

**Supplementary Materials**

**Dysfunctional Bone Marrow Endothelial Progenitor Cells are Involved in Patients with Myelodysplastic Syndromes**

**Authors:** Tong Xing^1,2^, Zhong-Shi Lyu^1,2^, Cai-Wen Duan^3^, Hong-Yan Zhao ^1^, Shu-Qian Tang^1^, Qi Wen^1^, Yuan-Yuan Zhang^1^, Meng Lv^1^, Yu Wang^1^, Lan-Ping Xu^1^, Xiao-Hui Zhang^1^, Xiao-Jun Huang^1,2^, Yuan Kong^1^*

**Correspondence to:** **Yuan Kong:** [**successky@163.**](mailto:successky@163.)**com**


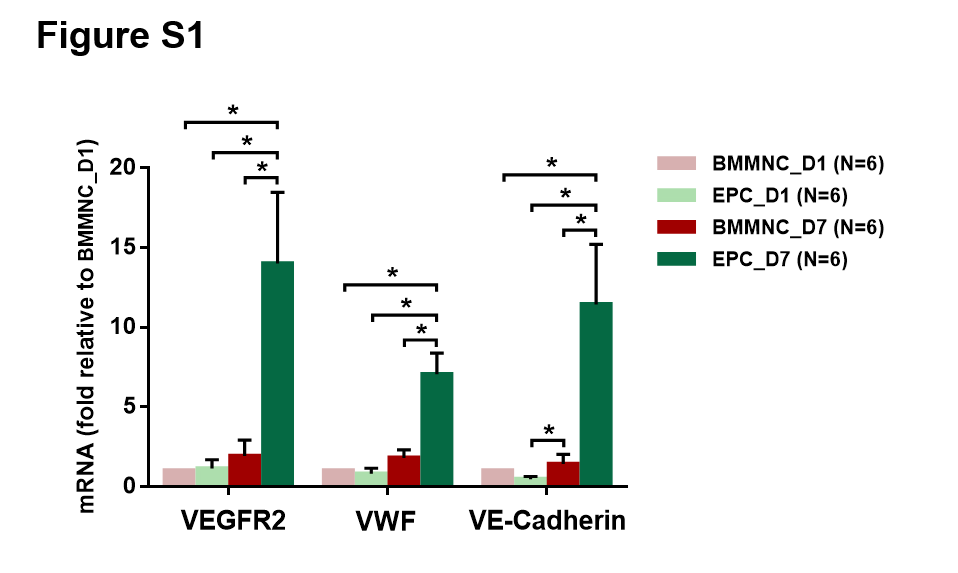
 **Figure. S1 Validation of EPC identity.** The mRNA levels of VEGFR2, VE-cadherin and VWF were analyzed by qRT-PCR between healthy donor BMMNCs cultured with EPC inducing media (EGM-2-MV-SingleQuots and 10% FBS) and without EPC inducing media (RPMI 1640 medium supplemented with 10% FBS) at day 1 (D1) and day 7 (D7). Statistical analyses were performed using Wilcoxon matched-pairs signed rank test. Data are presented as the means ± SEM (**P* ≤ 0.05). BMMNCs: Bone marrow mononuclear cells.

**Table S1. Clinical characteristics of MDS patients**

| **No.** | **Sex** | **Age,**  **y** | **Hgb,**  **g/L** | **Plts,**  **10^9^/L** | **ANC,**  **10^9^/L** | **Blast**  **%** | **Cytogenetics** | **Subtype** | **IPSS-R score** | **IPSS-R grade** |
| --- | --- | --- | --- | --- | --- | --- | --- | --- | --- | --- |
| 1 | M | 39 | 69 | 150 | 0.83 | 1 | del(20q) | MDS-MLD | 2.5 | L-MDS |
| 2 | M | 65 | 84 | 60 | 1.96 | 2 | (+11) | MDS-MLD | 3.5 | L-MDS |
| 3 | M | 60 | 72 | 85 | 2.26 | 4 | N | MDS-MLD | 3 | L-MDS |
| 4 | F | 57 | 98 | 318 | 1.69 | 1.5 | (+8, +15) | MDS-MLD | 3 | L-MDS |
| 5 | M | 37 | 57 | 140 | 0.62 | 1.5 | N | MDS-MLD | 2 | L-MDS |
| 6 | F | 69 | 71 | 35 | 0.9 | 1 | N | MDS-MLD | 2.5 | L-MDS |
| 7 | F | 57 | 106 | 27 | 0.34 | 2 | N | MDS-MLD | 1.5 | L-MDS |
| 8 | M | 38 | 53 | 56 | 0.14 | 2 | N | MDS-MLD | 2.5 | L-MDS |
| 9 | M | 45 | 41 | 229 | 0.74 | 0.5 | N | MDS-MLD | 2 | L-MDS |
| 10 | M | 62 | 46 | 602 | 1.07 | 2 | del(20q) | MDS-MLD | 2.5 | L-MDS |
| 11 | F | 29 | 80 | 44 | 0.18 | 2.5 | N | MDS-MLD | 3.5 | L-MDS |
| 12 | F | 42 | 82 | 56 | 1.8 | 2 | (+8) | MDS-MLD | 3.5 | L-MDS |
| 13 | F | 43 | 58 | 38 | 1.4 | 2 | N | MDS-MLD | 2.5 | L-MDS |
| 14 | M | 68 | 65 | 270 | 1.32 | 1.5 | N | MDS-MLD | 1.5 | L-MDS |
| 15 | F | 58 | 59 | 211 | 1.7 | 0.5 | (+8) | MDS-MLD | 3.5 | L-MDS |
| 16 | M | 57 | 50 | 14 | 1.12 | 6 | (−7) | MDS-EB | 7.5 | H-MDS |
| 17 | F | 64 | 38 | 118 | 3.02 | 18 | Complex | MDS-EB | 8.5 | H-MDS |
| 18 | F | 43 | 94 | 40 | 0.65 | 5 | N | MDS-EB | 4.5 | H-MDS |
| 19 | F | 67 | 82 | 20 | 0.32 | 16.5 | (+8) | MDS-EB | 7.5 | H-MDS |
| 20 | M | 49 | 70 | 24 | 0.03 | 6.5 | N | MDS-EB | 5 | H-MDS |
| 21 | M | 42 | 89 | 43 | 0.04 | 8 | Complex | MDS-EB | 8.5 | H-MDS |
| 22 | M | 24 | 83 | 42 | 0.91 | 10 | (+8, +11) | MDS-EB | 6 | H-MDS |
| 23 | M | 59 | 56 | 19 | 1.07 | 6 | N | MDS-EB | 4.5 | H-MDS |
| 24 | M | 55 | 92 | 42 | 0.27 | 14 | N | MDS-EB | 5.5 | H-MDS |
| 25 | M | 28 | 65 | 16 | 0.05 | 6 | N | MDS-EB | 5 | H-MDS |
| 26 | F | 51 | 72 | 94 | 3.63 | 6.5 | N | MDS-EB | 4 | H-MDS |
| 27 | M | 31 | 62 | 40 | 0.03 | 17 | Complex | MDS-EB | 10 | H-MDS |
| 28 | M | 41 | 50 | 38 | 0.59 | 12.5 | t(1;7) | MDS-EB | 9 | H-MDS |
| 29 | F | 68 | 78 | 151 | 0.38 | 8 | N | MDS-EB | 4 | H-MDS |
| 30 | F | 47 | 89 | 100 | 0.19 | 12 | Complex | MDS-EB | 8.5 | H-MDS |

**Abbreviations:** ANC, absolute neutrophil count (thousands/microliter); Hgb, hemoglobin; H-MDS, higher-risk MDS; IPSS-R: Revised International Prognostic Scoring System; L-MDS, lower-risk MDS; MDS-EB, myelodysplastic syndromes with excess blasts; MDS-MLD, myelodysplastic syndromes with multilineage dysplasia; Plts, platelets (thousands/microliter).

**Table S2. Antibody information**

| **Antibodies** | **Source** | **Identifier** |
| --- | --- | --- |
| V500 Mouse Anti-Human CD45 | BD Bioscience | 560777 |
| PerCP/Cyanine5.5 anti-human CD34 | BioLegend | 343522 |
| CD133/2 Antibody, anti-human | Miltenyi Biotec | 130-113-184 |
| PE Mouse Anti-Human CD309 (VEGFR-2) | BD Bioscience | 560494 |
| APC Annexin V | BioLegend | 640920 |
| 7-Amino-Actinomycin D (7-AAD) | BD Bioscience | 559925 |
| FITC anti-human CD34 Antibody | BioLegend | 343504 |
| PE-Cy7 Mouse Anti-Human CD3 | BD Bioscience | 557749 |
| BV510 Mouse Anti-Human CD8 | BD Bioscience | 563256 |
| PerCP/Cyanine5.5 anti-human IFN-γ | BioLegend | 502526 |
| PE Mouse Anti-Human IL-4 | BD Bioscience | 559333 |
| FITC anti-human IL-17A | BioLegend | 512304 |
| APC Mouse Anti-Human CD25 | BD Bioscience | 555434 |
| BV421 anti-human FOXP3 | BioLegend | 320124 |

**Table S3. Top 20 up and down regulated genes in higher-risk MDS BM EPCs than lower-risk MDS BM EPCs**

| **Gene** | **log_2_FoldChange** | ***P*-value** |  | **Gene** | **log_2_FoldChange** | ***P*-value** |
| --- | --- | --- | --- | --- | --- | --- |
| **Up regulated genes** | | |  | **Down regulated genes** | | |
| *AC016735.1* | 5.735385 | 4.37E-07 |  | *IGLV1-51* | -2.72399 | 4.91E-11 |
| *GTSF1* | 2.735294 | 3.71E-05 |  | *PRG3* | -5.5089 | 2.08E-08 |
| *SLITRK4* | 2.890701 | 0.000237 |  | *IGLC7* | -3.88307 | 4.62E-08 |
| *CCNA1* | 3.688518 | 0.00028 |  | *AFAP1L1* | -1.71417 | 8.55E-08 |
| *AC011043.1* | 2.027413 | 0.000313 |  | *IGHA1* | -2.63675 | 9.96E-08 |
| *FLT3* | 2.487448 | 0.000458 |  | *IGHGP* | -4.2747 | 1.04E-07 |
| *PIWIL4* | 1.636498 | 0.000527 |  | *IGHA2* | -1.96236 | 1.52E-07 |
| *TCTEX1D1* | 2.131847 | 0.000554 |  | *NFASC* | -1.78923 | 3.82E-07 |
| *APOBEC3B* | 1.797874 | 0.000589 |  | *ADTRP* | -3.81032 | 6.42E-07 |
| *MT1M* | 1.579657 | 0.001577 |  | *IGLC3* | -2.27095 | 7.82E-07 |
| *C3orf80* | 2.677886 | 0.001802 |  | *B3GNT7* | -2.51583 | 1.33E-06 |
| *CHRNA6* | 4.749994 | 0.001844 |  | *ANGPTL4* | -2.20767 | 2.17E-06 |
| *IL1B* | 2.104321 | 0.002339 |  | *IGHG3* | -2.58785 | 2.73E-06 |
| *PROM1* | 2.55931 | 0.002738 |  | *IGHV4-4* | -4.79739 | 2.75E-06 |
| *TSPOAP1* | 1.286821 | 0.002953 |  | *HBA1* | -4.05773 | 3.09E-06 |
| *AC002464.1* | 2.947937 | 0.003029 |  | *IGHD* | -3.59301 | 8.01E-06 |
| *TCEAL7* | 1.851452 | 0.003238 |  | *NXPH4* | -1.94832 | 8.21E-06 |
| *CBX2* | 1.740787 | 0.003263 |  | *COL23A1* | -3.97826 | 9.11E-06 |
| *IKZF2* | 1.239944 | 0.003555 |  | *SLC47A1* | -3.02838 | 1.17E-05 |
| *PCYT1B* | 4.502979 | 0.003635 |  | *CAMP* | -3.92198 | 1.91E-05 |

**Table S4. Top 20 up and down regulated genes in AML BM EPCs than higher-risk MDS BM EPCs**

| **Gene** | **log_2_FoldChange** | ***P*-value** |  | **Gene** | **log_2_FoldChange** | ***P*-value** |
| --- | --- | --- | --- | --- | --- | --- |
| **Up regulated genes** | | |  | **Down regulated genes** | | |
| *NCAM1* | 3.728861 | 1.12E-05 |  | *SELL* | -3.41395 | 5.96E-18 |
| *MT-ATP8* | 2.175184 | 2.90E-05 |  | *HDC* | -7.65131 | 1.00E-07 |
| *PROSER2-AS1* | 7.107244 | 7.48E-05 |  | *HLA-DRB5* | -3.76378 | 2.21E-06 |
| *DNM1* | 3.257067 | 0.000109 |  | *GPR174* | -2.515 | 4.57E-06 |
| *MYO7B* | 3.661201 | 0.00013 |  | *IL18RAP* | -3.81675 | 5.95E-06 |
| *AL713998.1* | 8.013028 | 0.000161 |  | *APOBEC3A* | -4.32147 | 6.35E-06 |
| *TRIM63* | 7.014626 | 0.000309 |  | *SELP* | -5.23768 | 9.93E-06 |
| *SMIM11A* | 3.718767 | 0.000323 |  | *C11orf21* | -1.81654 | 1.04E-05 |
| *RN7SL2* | 2.878153 | 0.000421 |  | *FCGR3B* | -4.14334 | 2.57E-05 |
| *SERPINB7* | 4.724996 | 0.000447 |  | *PPBP* | -4.60886 | 2.61E-05 |
| *COL23A1* | 5.492055 | 0.000494 |  | *HLA-DRB1* | -3.39812 | 2.67E-05 |
| *PSENEN* | 1.410605 | 0.000661 |  | *RHEX* | -3.83944 | 3.07E-05 |
| *AC125603.2* | 6.044281 | 0.000673 |  | *BEND4* | -4.765 | 3.63E-05 |
| *AC106865.1* | 6.830728 | 0.000768 |  | *TNNT3* | -6.52842 | 3.93E-05 |
| *AC005077.4* | 4.644503 | 0.000808 |  | *TSPAN32* | -1.98361 | 4.05E-05 |
| *ADTRP* | 2.932192 | 0.000846 |  | *HLA-DQA1* | -7.2352 | 4.64E-05 |
| *IGSF1* | 6.339027 | 0.001132 |  | *SKAP1* | -2.86866 | 4.72E-05 |
| *PDPN* | 3.682286 | 0.001244 |  | *KCNK17* | -4.02731 | 5.99E-05 |
| *KIF17* | 2.372966 | 0.001273 |  | *COL4A5* | -3.77789 | 6.64E-05 |
| *ADRA2C* | 2.742973 | 0.001298 |  | *ITGA2B* | -3.90607 | 7.44E-05 |

**Table S5. The primer sequences of genes used for qRT-PCR**

| **Gene** | **Forward primer sequence (5'-3')** | **Reverse primer sequence (5'-3')** |
| --- | --- | --- |
| *CXCL12* | CCAACGTCAAGCATCTCAAAAT | CACACTTGTCTGTTGTTGTTCT |
| *KITLG* | GTGGCAAATCTTCCAAAAGACT | CCATCTCGCTTATCCAACAATG |
| *NFKB1* | TATTTGAAACACTGGAAGCACG | CCGGAAGAAAAGCTGTAAACAT |
| *HAVCR2* | GAGTTACGGGACTCTAGATTGG | TGTTTTCTTCTGAGCGAATTCC |
| *CIITA* | TTGGGCAGAAAAGTCAGAAAAG | CTCAACGAGGAACTGGAGAAAG |
| *LGALS9* | CTGGACAGATGTTCTCTACTCC | ACCACAGCATTCTCATCAAAAC |
| *CASP2* | AGGACATCATCACCTTGGAAAT | AAGTTGAGGAGTTCCACATTCT |
| *CASP3* | CCAAAGATCATACATGGAAGCG | CTGAATGTTTCCCTGAGGTTTG |
| *BAX* | CGAACTGGACAGTAACATGGAG | CAGTTTGCTGGCAAAGTAGAAA |
| *TP53* | TTCCTGAAAACAACGTTCTGTC | AACCATTGTTCAATATCGTCCG |
| *CDKN1A* | GATGGAACTTCGACTTTGTCAC | GTCCACATGGTCTTCCTCTG |
| *CCNE1* | TTGTGTCCTGGCTGAATGTATA | AAGGAAATTCAAGGCAGTCAAC |
| *MCL1* | GATGTGAAATCGTTGTCTCGAG | GAAATGAGAGTCACAATCCTGC |
| *VE-cadherin* | AAAGAATCCATTGTGCAAGTCC | CGTGTTATCGTGATTATCCGTG |
| *VWF* | CCTGTTACTATGACGGTGAGAT | CATGAAGCCATCCTCACAGTAG |
| *VEGFR2* | GGAGCTTAAGAATGCATCCTTG | GATGCTTTCCCCAATACTTGTC |
| *18S* | GTAACCCGTTGAACCCCATT | CCATCCAATCGGTAGTAGCG |
